# Phenological niches and the future of invaded ecosystems with climate change

**DOI:** 10.1093/aobpla/plu013

**Published:** 2014-03-31

**Authors:** Elizabeth M. Wolkovich, Elsa E. Cleland

**Affiliations:** 1 Biodiversity Research Centre, University of British Columbia, Vancouver, BC, Canada; 2 Arnold Arboretum, Harvard University, Boston, MA, USA; 3 Organismic & Evolutionary Biology, Harvard University, Cambridge, MA, USA; 4 Division of Biological Sciences, University of California – San Diego, La Jolla, CA, USA

**Keywords:** Alien or exotic species, climate change, invasions, non-native, phenology, plasticity, temperate systems.

## Abstract

In recent years, research in invasion biology has focused increasing attention on understanding the role of phenology in shaping plant invasions. Multiple studies have found non-native species that tend to flower distinctly early or late in the growing season, advance more with warming or have shifted earlier with climate change compared with native species. This growing body of literature has focused on patterns of phenological differences, but there is a need now for mechanistic studies of how phenology contributes to invasions. To do this, however, requires understanding how phenology fits within complex functional trait relationships. Towards this goal, we review recent literature linking phenology with other functional traits, and discuss the role of phenology in mediating how plants experience disturbance and stress—via climate, herbivory and competition—across the growing season. Because climate change may alter the timing and severity of stress and disturbance in many systems, it could provide novel opportunities for invasion—depending upon the dominant climate controller of the system, the projected climate change, and the traits of native and non-native species. Based on our current understanding of plant phenological and growth strategies—especially rapid growing, early-flowering species versus later-flowering species that make slower-return investments in growth—we project optimal periods for invasions across three distinct systems under current climate change scenarios. Research on plant invasions and phenology within this predictive framework would provide a more rigorous test of what drives invader success, while at the same time testing basic plant ecological theory. Additionally, extensions could provide the basis to model how ecosystem processes may shift in the future with continued climate change.

## Abstract (precis)

We review recent evidence that non-native and invasive plant species may have distinct timings of their seasonal life history characteristics (such as date of leaf out or flowering, that is, their phenology) that allow them to establish in new communities. In particular we examine how invasions may be bolstered by the longer growing seasons associated with climate change. Based on our current of plant phenology and growth strategies—especially rapid growing, early-flowering species versus later-flowering species that make slower-return investments in growth—we project optimal periods for invasions across three distinct systems under current climate change scenarios.

## Introduction

Understanding the forces that allow species to invade established communities is a central goal of ecology (Elton 1958) and critical to mitigating impacts of invasive species (Levine *et al.* 2003). Mechanistic models of community assembly have helped develop frameworks for predicting when and where invasions are likely to occur (e.g. Shea and Chesson 2002); however, numerous factors may influence invasion success, including competition with established species for limiting resources (MacArthur 1970; Tilman 1982, 1988), interactions with higher trophic levels (Keane and Crawley 2002; Colautti *et al.* 2004) and processes associated with environmental variability (Chesson and Warner 1981; Chesson 1986). Further, climate change may facilitate invasion by non-native species (Dukes 2011). While many studies have focused mechanistically on direct positive effects of warming or resource enhancement on invasive species (Bradley *et al.* 2010), there is growing recognition that climate change could facilitate invasions because of the distinct phenology or phenological sensitivity of non-native species (Willis *et al.* 2010; Wolkovich *et al.* 2013).

**Figure 1.**
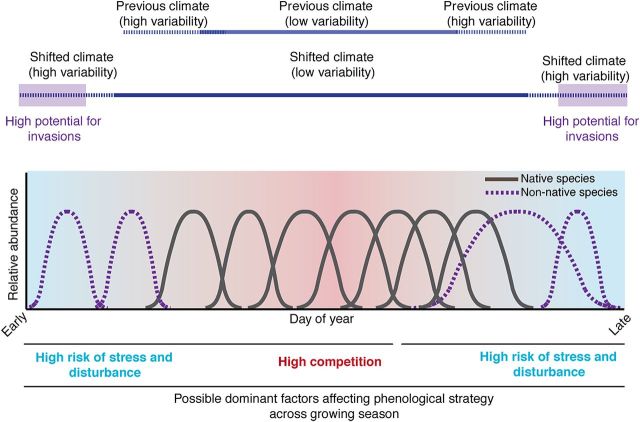
Basic invasion theory, built on limiting similarity theory, suggests that species should invade during times when most other species are inactive (vacant phenological niche; see Wolkovich and Cleland 2011). Here we show idealized niche diagrams for four non-native species (purple, dashed-line distributions) and seven native species (grey distributions) in a hypothesized simple mesic temperate system where temperature limits viable periods for plant growth. Across the growing season, variation in stress, disturbance and competition may dictate the optimal phenological strategy, with early-active and late-active species experiencing lower competition but also more variable temperatures, in the mid-season community flowering peaks (see Fig. 2), and thus we expect that mid-season active species may be strong competitors for many resources. With climate change extending viable periods for plant growth (dark blue lines), non-native species with highly plastic phenologies may have an increased opportunity for invasion at the start and end of the growing seasons in temperate mesic systems. As reviewed in Wolkovich and Cleland (2011), there are several major ways in which species may exploit such vacant phenological niches. Species that can track the start of the season closely may exploit even very small vacant niches in the early season via priority effects. Additionally, climate change—by extending growing seasons in many systems—may increase vacant niche space at the start and end of the growing season, possibly allowing for invasions early and late in the season. Non-native species with early phenology and rapid growth strategies may succeed either early or late in the season, while species with greater phenological niche breadth (e.g. longer flowering period) may succeed late in the season.

Theories regarding fluctuating resources (Davis *et al.* 2000) and ‘windows of invasion opportunity’ (Drake *et al.* 2006; Caplat *et al.* 2010) suggest that seasonal phenology—the timing of life history events—may play a critical role in invasions (Wolkovich and Cleland 2011). Models of invasion success that hinge on phenology build from the concept of a temporal niche (Fig. 1)—that time is a fundamental axis by which species may partition resource use (Gotelli and Graves 1996), reduce interspecific competition and thus promote coexistence (Chesson and Huntly 1997). Extensions of this basic niche theory have suggested how such distinct phenologies may result in a competitive advantage for non-native species (Godoy *et al.* 2009), especially in areas with shifting growing seasons due to climate change. If native species do not accurately and rapidly track shifting climate, then climate change may produce phenological vacant niches. In brief, such vacant niches may then promote invader success (i) directly (i.e. an invader occupies the open niche space) or in concert with (ii) early-season priority effects, via (iii) invader plasticity, where non-native species track climate shifts more closely than native species, or (iv) greater niche breadth (see Fig. 1 and Wolkovich and Cleland 2011). Alongside these theoretical developments, a growing body of research focused on plants has found phenological differences, especially in leafing and flowering times, between non-native and native species. Several studies have found that especially early (McEwan *et al.* 2009; Wilsey *et al.* 2011; Throop *et al.* 2012; Wainwright *et al.* 2012) or late (Godoy *et al.* 2009; Fridley 2012; Paquette *et al.* 2012; Pearson *et al.* 2012) phenologies may aid the success of non-native species, while more recent work suggests that non-native species may be the major drivers of longer growing seasons in North America (Wolkovich *et al.* 2013). Here we build on current theoretical perspectives and empirical work to develop predictions of how phenology may enhance plant invasions with climate change. Our review begins first with the role of phenology in avoiding or mitigating disturbance, stress and competition. Next, we review recent literature on plant functional traits to highlight evidence for a fundamental trade-off between flowering phenology and the return rate of growth investments, which may impact how climate change and phenology affect invasions. Considering projected scenarios of climate change, we make mechanistic predictions for when during the growing season across three major ecosystem types vacant phenological niche space may promote invasions, and consider current evidence for our predictions. We close by reviewing major questions whose answers would improve predictions of future invasions via phenology.

### A note on terminology

Given the debate over terminology in invasion biology (Colautti and Richardson 2009), we wish to be clear about our definitions. We use ‘non-native’ to refer to any species established outside of its home range; such a distinction between native and non-native is important because non-native species have evolved in a different community than the one into which they have been introduced. Thus, they may exhibit differing strategies and trade-offs than native species. We use the term ‘invasive’ for non-native species with a detrimental impact in their introduced community (following Mack *et al.* 2000). Finally, ‘invade’ and ‘invasion’ refer to the introduction of species, whether they are eventually invasive or not.

## Phenological Strategies

### Phenology within plant life history theory

Phenology is an important component of plant life history theory (Al-Mufti *et al.* 1977; Grime 1977; Stanton *et al.* 2000) affecting both biotic constraints (e.g. competition, herbivory, pollination) and abiotic constraints (e.g. frost, drought) on plant performance. Extensive work over the past several decades has focused on how biotic interactions are informed by phenology, including competition (e.g. Rathcke 1988; van Schaik *et al.* 1993) and mutualisms (e.g. Brody 1997), while recently the balance of studies has shifted towards a more abiotic focus with climate change (e.g. Inouye 2008; Miller-Rushing and Primack 2008).

Plant strategy theory has generally focused on how both abiotic and biotic factors affect acquisition, allocation and loss, often extrapolating into a focus on how well plants handle competition, stress and disturbance (Grime 1977). Stress, as generally defined, does not lead to major tissue loss while disturbance does—thus the best definition of stress versus disturbance is often species and location specific (Craine 2009). For example, in temperate systems, both frost (abiotic) and herbivores (biotic) may act as a disturbance around which plants must balance their leafout timing. The major difference between the plant's ability to adapt to these abiotic and biotic constraints arises via the feedbacks possible with biotic factors (e.g. herbivores may adjust, within their own set of climatic limits, to match earlier leafout). In contrast, abiotic constraints on plant growth (e.g. frost) are unlikely to be impacted by plant phenology. Thus, in most systems where abiotic factors have been relatively stationary across years—in timing and variability especially—we expect plants to have adjusted their strategies to these system properties. Further, given temporal variation in the abiotic and biotic environment (i.e. across the growing season and across years), we expect phenology to be a major axis along which plants structure their overall life history strategies (Grime 1977). Indeed, recent research in the expanding field of functional plant traits suggests that phenology may be tied to a suite of other traits producing several major phenological strategies.

### Trait correlations with phenology

A review of the functional traits literature (Table 1) highlights a strong axis for flowering phenology where earlier flowering is associated with a suite of traits related to rapid return on investment, while later flowering is often associated with the reverse. This axis makes sense when considering how stress, disturbance and competition vary across the growing season in many systems (Fig. 1): early in the season when abiotic stress and disturbance are high, but competition low, an early-flowering, rapid-growth and comparatively low-investment strategy allows species to grow and reproduce quickly before periods of strong competition begin. Such a strategy may also make some loss of tissue to environmental disturbance early in the season less detrimental if rapid growth allows rapid replacement of tissue. While it may seem obvious that earlier flowering would require a quicker return on investment, many perennial species use resources from previous years for the current-year's flowering, and thus this correlation is not automatic (Muller 1978). Further, such a trade-off is seen across both herbaceous and woody species (Table 1). In contrast to early-flowering species, species that flower later in the season must survive high competition throughout the mid-season and thus traits that allow more efficient access to, transport and use of resources would be key. Loss of tissue to disturbance in such a strategy, however, may impart a relatively higher cost, as regrowth would be much slower. This major phenological trait axis—of early and fast versus later and slower—has been noted by many researchers (e.g. Lechowicz 1984; Sun and Frelich 2011), but an additional strategy may be viable in the late season. It seems that some species may also exhibit a rapid-growth and low-investment strategy in the late season (Sun and Frelich 2011); however, as this has been noted less often, we do not consider it extensively.

**Table 1.** Current research suggests one major axis by which phenology co-varies with other traits: earlier flowering (and in some cases, earlier leafing) is often associated with traits related to quicker returns on investments (faster growth rates, shallower roots, etc.) while later-flowering species show traits associated with slower returns on investments (slower growth rates, greater heights, deeper roots, etc.). Studies characterizing this trade-off are presented above the double line, while additional studies are shown below. We reviewed the literature using ISI Web of Science and the following search: Topic=(phenolog*) AND Topic=(plant*) AND (functional trait*) Refined by: Document Types=(ARTICLE) AND Web of Science Categories=(ECOLOGY), which returned 79 papers. Of these we included studies that documented phenology and at least one other trait for multiple species. Studies were excluded if they only studied animal guilds or if they focused on selection within a single plant species. Additionally, leaf lifespan was omitted as a measure of phenology if it was simple evergreen/deciduous (as this represents more leaf lifespan than phenology). We included several additional studies that we encountered in the process of writing the manuscript. ‘Flowering date’ includes flowering date, peak flowering date and flowering onset date; SLA=specific leaf area.

Phenological trait	Other trait(s) studied	Relationship	Plant functional group(s)	Location(s) and reference(s)
Flowering date	Max height	Positive (earlier, smaller)	Herbaceous species	Mediterranean old field in Israel (Hadar *et al.* 1999); semi-natural grasslands in France (Louault *et al.* 2005; Vile *et al.* 2006); southeastern Sweden (Bolmgren and Cowan 2008); Tibetan plateau (Du and Qi 2010); eastern North America (Sun and Frelich 2011); mountain meadows in Italy (Catorci *et al.* 2012)
Flowering date	Max height	Positive (later, taller)	Mixed woody and herbaceous	Ponderosa pine forest (Laughlin *et al.* 2010)
Flowering date	Seed size	Positive (later, larger)	Herbaceous species	Semi-natural grassland in France (Vile *et al.* 2006)
Flowering date	Seed size	Negative (earlier, smaller)	Mixed woody and herbaceous	Indiana (USA) dunes (Mazer 1989)
Flowering date	Growth rate	Negative (earlier, faster)	Herbaceous species	Eastern North America (Sun and Frelich 2011)
Flowering season	Rooting depth	Positive (later, deeper)	Herbaceous species	Patagonian Steppe (Golluscio and Sala 1993); Mediterranean annual grassland, CA, USA (Gulmon *et al.* 1983); Tibetan plateau (Dorji *et al.* 2013)
Length of growing season	Rooting depth	Positive (later, deeper)	Mixed	Semi-arid woodland in Australia (Campanella and Bertiller 2008)
Flowering date	Generation time	Positive (later, longer)	Herbaceous species	Semi-natural grassland in France (Vile *et al.* 2006)
Flowering date	SLA	Negative (earlier, thinner)	Herbaceous species	Semi-natural grassland in France (Vile *et al.* 2006)
Length of growing season	SLA	Negative (longer, thicker)	Mixed	Semi-arid woodland in Australia (Campanella and Bertiller 2008)
Leafout date	Diameter of spring vessels and/or greater numbers of narrow-diameter vessels	Positive (later, larger)	Trees	Northern North American forests (Lechowicz 1984)
Flowering date	Leaf tissue density	Positive (later, greater)	Herbaceous species	Tallgrass prairie in Kansas, USA (Craine *et al.* 2012*b*)
Flowering date	Grazing tolerance	Negative (later, tolerant)	Herbaceous species	Mediterranean old field in Israel (Hadar *et al.* 1999)


Leaf flushing date	SLA	Positive (later, thinner)	Trees	Savannah/Cerrado in Brazil (Rossatto *et al.* 2009)
Length of leaf season	Leaf size	Positive (later, larger)	Mixed woody and long-lived perennial species	High-elevation Mediterranean woodland, Morocco (Navarro *et al.* 2010)
Flowering date	Seed size	Negative (later, smaller)	Herbaceous species	Southeastern Sweden (Bolmgren and Cowan 2008)
Flowering date	Seed size	Bimodal (early and late flowering had small seeds, mid-season was mixed)	Herbaceous species	Mountain meadows in Italy (Catorci *et al.* 2012)
Mixed	Morphology, leaf thickness, photosynthetic pathway, life history, seed biology	Complex (multivariate)	Mixed woody and herbaceous	Semi-arid woodland, Australia (Leishman and Westoby 1992); northeast China (Wang and Ni 2005)
Length of growing season	Origin	Invading species had longer, later-growing seasons	Mixed woody and herbaceous	Germany (Kuester *et al.* 2010)

Examining phenology as one trait within a complex network of correlated traits raises an important issue of considering when phenology is a major trait on which selection acts, versus only linked to a more critical trait. For example, flowering time is often associated with seed size (Table 1), and teasing out how much phenology or seed size is constrained by the other remains a puzzle, with correlations varying by clade and study (Mazer 1989; Bolmgren and Cowan 2008). Thus, phenology may be structured strongly by selection on it directly, via links with other traits, or shaped by evolutionary history (see Lechowicz 1984; Ollerton and Lack 1992, and see below). If, however, phenology is a major trait structuring life history strategies, then given a relatively stationary abiotic and biotic environment we would expect each species to optimize its phenology for that environment. Given sufficient time and species dispersal, we would also predict that communities would contain a suite of phenological strategies that take up most available resources across the growing season.

## Climate Change, Phenology and Invasions: Predictions

Climate change has altered the climate of most ecosystems globally such that they cannot be considered stationary (that is, to have consistent climate means with some stochastic variation around those means; see Betancourt 2012). Non-stationarity could change the optimal phenological strategy—both in absolute timing and in flexibility in this timing—leaving native species less well-adapted to their current environment and providing an opportunity for invasions. Understanding how phenology may intersect with plant invasions requires an explicit temporal model of how competition, stress and disturbance vary across growing seasons and with climate change. Such a model should make basic but testable predictions about when (within a growing season and over longer timescales), in which systems and how phenology may contribute to invader success. We lay out general predictions below, but focus on how temperature increases and precipitation change may affect species invasions. Given this focus, we consider predominantly three types of systems that differ in the dominant abiotic controls over phenology: temperate mesic systems (temperature control), temperate grasslands (temperature and precipitation control) and semi-arid systems (precipitation control). These systems provide useful contrasting examples of how competition, stress and disturbance may interact with phenology to influence community assembly and invasions. We base our predictions on recent climate change projections (Knutti and Sedlacek 2013), trait correlations and plant strategies related to phenology (Table 1 and above), and more generally to invasion. Specifically, we assume (i) that across space and time non-native species may invade environments of relatively high stress and disturbance, but low competition (e.g. Rejmánek 1996; Gelbard and Belnap 2003), up to a point, (ii) as there is also evidence that non-native plant species rarely occupy the most climatically stressful environments (Rejmánek 1989). Additionally, (iii) invaders are often more plastic and thus may adjust to new conditions quickly (Funk 2008; Hierro *et al.* 2009; Davidson *et al.* 2011; Wainwright and Cleland 2013). We thus predict temporal opportunities for invasions in periods of relatively high stress and disturbance, but low competition, and discuss how and when climate change may alter these opportunities.

### Predictions: early season

**Figure 2.**
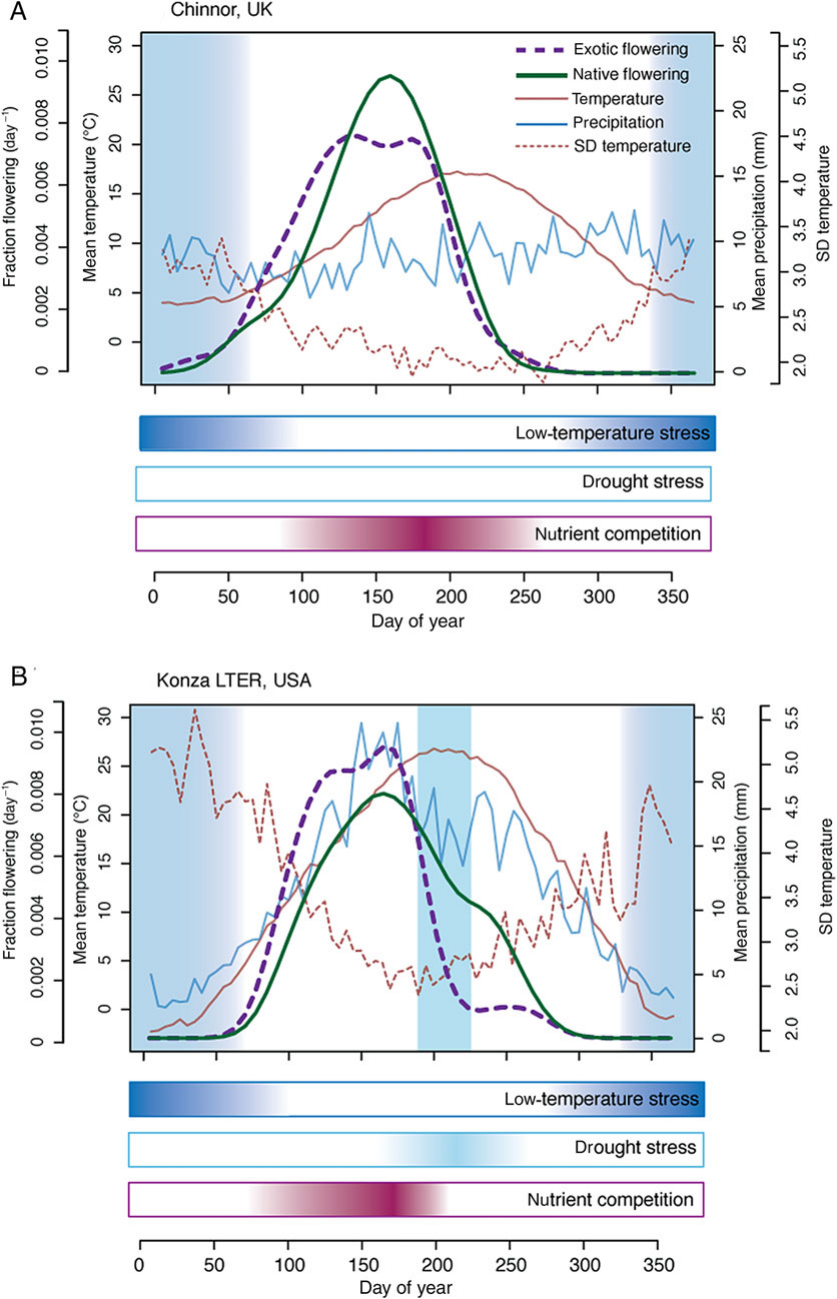
Flowering of non-native and native species within a community varies across habitats. This variation in flowering patterns may be strongly driven by climate differences between systems, which impact the various flavours of stress, disturbance and competition that plants experience. Mesic temperate systems such as Chinnor (UK, A) are often defined by temperature (darker blue shading), while other systems such as the tallgrass prairie of Konza (Kansas, USA, B) may have variable drivers across the growing season. In both systems, temperature sets the beginning and end of the season and, as such, early-season species show strong sensitivity to temperature (Cook *et al.* 2012; Craine *et al.* 2012*b*). In Konza, a consistent mid-season drought, however, coincides with a decrease in the number of species flowering at that time (Craine *et al.* 2012*b*). We assume that temperature <5 °C limits development, as this is the temperature at which many cell processes slow down dramatically or stop (Larcher 2003) and, further, is the suggested lower threshold temperature for tissue growth and development globally for alpine trees (Korner 1998). Standard deviation (SD) of temperature and the coefficient of variation (CV) of precipitation are given as pentad (5-day) averages. Flowering data are species averages from NECTAR (Wolkovich *et al.* 2012*b*), climate data for Chinnor were taken from GHCN UK000056225 and cover 1853–2001, while climate data for Konza were taken from GHCN USC00144972 and cover 1893–2010.

Across systems with distinct growing seasons, the early season often represents a period of relatively high stress and disturbance but low competition (Fig. 2)—as most species slowly begin to reactivate tissues and grow. Climatically, the early portion of a temperate growing season is signalled by a rise in temperature. This rise, however, is correlated strongly with increased variability in temperatures (Fig. 2), resulting in high stress and possible disturbance for plants active during this period, and—relatedly—low competition. Plant species active in the early season may risk tissue loss to frost damage or other extreme temperature swings present in the spring (Linkosalo *et al.* 2000; Augspurger 2009), or to greater herbivore damage (Lechowicz 1984), as species may be more apparent to herbivores in the early season (Brody 1997) and have less-defended tissues (van Asch and Visser 2007). Early-season phenologies, however, also benefit from reduced competition. Across habitats, few species leaf and flower early (Fig. 2) yielding lower competition for soil and light resources and for pollinators (Mosquin 1971). Additionally, competition with microbes for soil nutrients may be especially low as in many systems the soil microbial community turns over with warm spring weather, producing a flush of soil nutrients (Zak *et al.* 1990).

Early phenologies of non-native species may thus succeed through two major mechanisms independent of climate change. First, non-native species that are active early in the growing season may be particularly successful because they have the opportunity to preempt space and soil resources, grow quickly and shade later-active species (Weiner 1990; Wilsey *et al.* 2011; Wolkovich and Cleland 2011). This type of asymmetric competition could create ‘seasonal priority effects,’ one mechanism by which non-native species could establish and rise to dominance in a new community (Dickson *et al.* 2012; Wainwright *et al.* 2012). Second, invaders may succeed via an early-season enemy release mechanism. In invasion biology the enemy release hypothesis suggests that non-native species could be less susceptible to herbivory than native species due to a lack of specialist herbivores (Keane and Crawley 2002; Liu and Stiling 2006). Thus, if the early season is a critical period of susceptibility to herbivory (Feeny 1970; Barbehenn *et al.* 2013), this may also be the critical time for invaders to benefit from herbivore release. This suggests a mechanism by which non-native species could break the risk–benefit trade-off of early phenology experienced by native species, thereby giving them a special advantage from early-season phenology.

**Figure 3.**
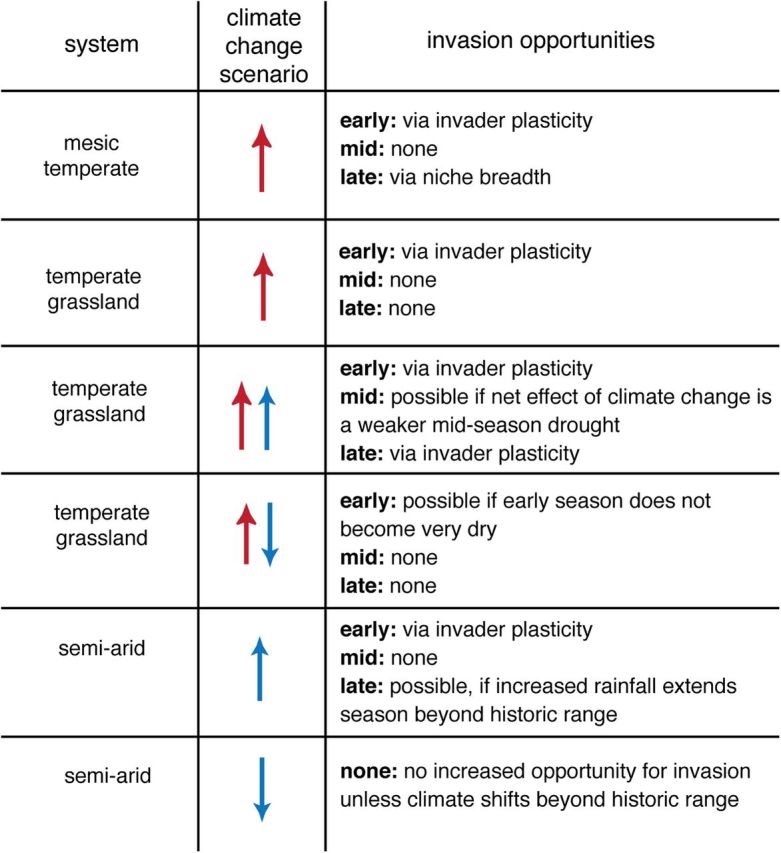
Predictions for how climate change may promote invasions vary across the growing season, across systems with differing dominant climate regimes, and by how climate shifts (red arrows refer to temperature increases, while blue arrows refer to precipitation increases or decreases). Here we consider three major systems and how dominant climate drivers are projected to shift with climate change, based on recent models (Knutti and Sedlacek 2013). Because models of precipitation shifts are often divergent (Knutti and Sedlacek 2013), for systems with precipitation control we consider increases or decreases in precipitation. In all systems an increase in the dominant climate factor that controls the start of the season may increase invasions by species that can track this shift closely (invader plasticity). Because we suggest that successful invasions are rare in times of very high resource competition and extremely high climatic stress and disturbance, we do not predict invasions during periods when competition is already high (mid-season of many systems) or when climate change increases drought stress (declines in precipitation in semi-arid systems or during mid-season drought in grasslands). When climate change pushes systems far beyond their historic climate regimes, however, native species may be pushed well away from their optimal climate, and we may see an increase in invasions. See the main text for further details, including background assumptions leading to predictions.

Climate change may additionally promote early-season phenologies and provide a mechanism whereby non-native species with early-season phenologies are especially successful. Recent increases in spring temperatures in the temperate biome (at least partially associated with increases in greenhouse gases; see Trenberth and Josey 2007) have been studied extensively. Most studies find that spring temperatures have increased as much or more than temperatures in other seasons (Cohen *et al.* 2012), meaning this is a season of especially high non-stationarity in climate (Fig. 1). Predictions for precipitation-limited systems are more variable but include options for increased total and increased variability in early-season rainfall (Trenberth and Josey 2007; Knutti and Sedlacek 2013). Such high variability may make it an optimum period for invasion of other species for several reasons. First, such high non-stationarity should mean native species are being pushed far away from the previous long-term climate means to which their phenology should be adapted. Next, if non-native species have higher phenological plasticity (e.g. Wainwright and Cleland 2013), they may more closely track shifting climate than native species. Early flowering is also often correlated with plant traits related to rapid growth. Non-native species exploiting an early-season vacant niche may thus grow rapidly and take up much of their needed resources to complete reproduction before competition with native species effectively sets in (Fig. 3).

### Predictions: mid-season

In most systems, the mid-season represents the period when the greatest number of species flower (e.g. Fitter *et al.* 1995; Aldridge *et al.* 2011), and work to date using community datasets suggests that this is true for both native and non-native species (Wolkovich *et al.* 2013). This is perhaps not surprising as in most systems the mid-season represents a period of relatively low stress due to the physical environment and high abundance of pollinators. This high abundance of species in flower, however, means it is also the period of high competition for resources (Fig. 2) and thus we generally predict few invasions driven by phenology mid-season (Fig. 3).

In some systems with precipitation control, however, the mid-season often has a reduced period of plant competition associated with a mid-season drought (Fig. 2). Temperate grasslands, for example, are generally characterized by mid-season droughts when the highest temperatures coincide with low precipitation, drying soils and fewer species that initiate flowering during this period (e.g. Craine *et al.* 2012*b*). This reduced competition could result in an opportunity for invasion. However, because it also generally represents a period of extremely high drought stress, we do not predict invasions mid-season generally. Increases in mid-season precipitation—especially those outside historical ranges—may, however, provide a novel vacant niche. Many early-flowering species appear to end flowering well before drought onset and, depending on the phenological cues they use for flowering onset and end, may thus not be able to exploit greater mid-season precipitation because they have adapted primarily to avoid the mid-season drought (Craine *et al.* 2012*b*, 2013). Native species flowering during the mid-season may have trade-offs between drought tolerance and competitive abilities for other resources (Craine *et al.* 2013), which may make them less successful at exploiting increased mid-season precipitation. We speculate that, under this scenario, increased mid-season precipitation (that is not offset by higher temperatures) may promote mid-season invaders with climate change.

### Predictions: late season

Climatically, in many systems the end of the growing season mirrors the beginning (Fig. 2), but plant phenology differs strongly. For example, while in both temperate mesic and grassland systems plant leafing and flowering (Fig. 2) generally tracks closely the rise in spring temperatures around 5 °C (Larcher 2003), community flowering curves generally end at least one month before temperatures return to 5 °C. The reasons for this may be simply related to physiological constraints: species must flower well before the end of the season in order to have enough time to fruit and set seed. A balance between risk and investment may also be important: as mean temperatures drop, temperature variability climbs (Fig. 2), just as low spring temperatures are also correlated with higher temperature variability. In contrast to the spring, however, later in the season almost all species have made a heavy investment in growth and reproductive tissues and loss to frost may pose an even greater threat than frosts in the spring; thus plants may be especially conservative, even those with a ‘late and fast’ phenological-growth strategy. This risk/investment balance may explain why many species often have plastic spring phenologies based on temperatures that are flexible across habitats, but static fall phenologies based mainly on photoperiod cues (Howe *et al.* 2003). The result is that, in many systems, the end of the growing season is a period of generally climatically high stress and disturbance, but low competition. Without climate change we expect that most native species have adapted towards the optimal time to senesce based on their risk–investment strategy and there may be little opportunity for invasion.

With climate change, however, we expect pronounced opportunities for invasion late in the growing season when climate change extends the end of the season (Fig. 3), as native species may be constrained by their evolutionary history, and cues associated with the end of the growing season (i.e. photoperiod). Thus, non-native species with greater phenological niche breadth (either via a generally static longer growth or flowering period, or via greater flexibility to extend their phenology resulting in greater niche breadth) may be able to exploit this late-season vacant niche. This should apply to all systems where the late season is a period of relatively high stress and disturbance but low competition—up to a point. In many systems where both temperature and precipitation shape growing season dynamics, the late season can have high drought stress; we do not expect significant invasion during this window because non-native species may not be as adapted to high drought stress compared with the native species in these systems (Alpert *et al.* 2000).

## Role of Climate Change in Phenologically Mediated Invasions: Evidence to Date

### Results: early season

Work to date supports evidence for early-season invasions, which are linked to climate change in several temperate mesic systems (Wolkovich *et al.* 2013) and linked to seasonal priority effects in semi-arid systems (Dickson *et al.* 2012; Wainwright *et al.* 2012). Additionally, research on one non-native understorey shrub species (Xu *et al.* 2007) suggests a role for seasonal priority effects in temperate forest systems. Results in temperate grasslands, however, do not show a strong link between early phenology and invasion (Wolkovich *et al.* 2013). Across temperature-controlled systems, however, early-season species, whether native or non-native, also tend to be the most sensitive to temperature (Cook *et al.* 2012; Wolkovich *et al.* 2012*a*). Relatedly, multiple studies using various methods now show that in many mesic temperate biomes, invaders are highly sensitive to temperature—tending to advance their phenology significantly more than native species (Willis *et al.* 2010; Wolkovich *et al.* 2013), though, again, this does not appear to be the case in temperate grasslands (Wolkovich *et al.* 2013). Moving forward, accurate predictions of phenologically mediated invasions will require teasing out exact mechanisms. Thus, future research with climate change in temperate biomes will need to quantify how much invasion success occurs because of flexibility in phenology (i.e. the trait of the invader leads to success) versus via open niche opportunities present in the early season due to non-stationarity with climate change (i.e. the system is open to invasion in the early season), or a mix of the two scenarios.

### Results: mid-season

Several North American studies have documented declines in native species alongside shifts with climate change in mid-season drought periods, generally showing that the drought period may be extending or becoming more pronounced (Aldridge *et al.* 2011), and multiple authors have postulated that this period will result in a vacant niche for invaders (Sherry *et al.* 2007; Aldridge *et al.* 2011). To date, however, no studies (of which we are aware) have shown invaders occupying these mid-season drought periods, while in contrast two studies of phenology at Konza Prairie LTER have found a decline in non-native flowering coinciding with the mid-season drought (Craine *et al.* 2012*b*; Wolkovich *et al.* 2013). Further, work on previous extreme droughts has generally documented a shift in native species composition, but not invasions (Weaver and Albertson 1936). These patterns are based on findings in North American prairie systems; however, far more work is needed additionally to understand if this period is occupied by invaders in other systems or is possibly too stressful. This represents an area where predictions are difficult for several reasons: (i) understanding and modelling how species respond to moisture has proven far more difficult than modelling temperature responses (Crimmins *et al.* 2011; Wolkovich *et al.* 2013), (ii) work to date suggests that phenological responses to drought are highly variable between different species (Jentsch *et al.* 2009; Prieto *et al.* 2009), and (iii) projections of how precipitation and droughts will shift in the future are some of the most uncertain of all climate change forecasts (Knutti and Sedlacek 2013).

### Results: late season

Recent studies of plant invasions, especially in eastern North American forests, suggest that non-native and invasive species may successfully exploit a late-season vacant niche via greater niche breadth in temperate biomes—which may be linked to climate change. A pervasive non-native understorey species in the Ohio River Valley, *Lonicera maacki*, stays green later than any native understorey species (Becker *et al.* 2013). Similarly, the invasive tree *Acer platanoides* also stays green later than one studied North American congener (Paquette *et al.* 2012). Further, recent work suggests that these non-native species may not play by the same risk–investment rules as native species in the fall. A study of several dozen North American non-native species from the understorey confirms that these species consistently senesce in the fall later than native species (Fridley 2012). Fridley (2012) also found that many of these species remain green until the first major frost and thus lose their leaves to frost versus plant-induced senescence. This should be a major cost to the plant—as most species resorb nutrients well before the first frost (Lambers *et al.* 2008). Further work showed that the longer leaf lifespan of the non-native species enabled a greater time-integrated nutrient-use efficiency (Heberling and Fridley 2013), and, coupled with high rates of nutrient uptake the following spring, provides a mechanism by which the unique phenology of non-native species, compared with the native community, could promote invasion.

If these non-native species in eastern North America do gain a large benefit from occupying an open late-season temporal niche, even while losing tissue to frost, then climate change could further increase their success. In many habitats, fall temperatures are rising more quickly than even spring temperatures (Cohen *et al.* 2012), and are expected to continue to rise—further extending the open niche space at the end of the season, which already appears temporally much greater than the early season (Fig. 2). Thus, the late season appears to be a period of very low plant competition and is often also when microbial communities turn over (Bardgett *et al.* 2005), suggesting that species which can remain active until the end of the season may have access to a large available resource pool.

Across systems, however, autumnal shifts in climate and phenology are still relatively unstudied compared with spring. More work is needed to understand where, and by how much, mean fall temperatures are shifting in comparison to other seasonal temperature shifts and how species are adjusting their late-season phenological events. In particular, there is very little work from the late season in grasslands, where the combination of shifts in mid-season droughts alongside shifted fall temperatures may create novel climates and, possibly, novel opportunities for invasion. Alternatively, systems with mid-season droughts may have reduced opportunities for invasion if such droughts—over the long term—have favoured more variable phenological strategies (e.g. species flower very late to avoid stress of mid-season drought; see Craine *et al.* 2012*a*) compared with simple temperature-controlled systems, which have been noted to have far greater late-season empty phenological space compared with grasslands (Craine *et al.* 2012*a*).

## Major Questions

Recent research connecting phenology and invasions has clearly provided support for the idea that invaders may benefit from phenological vacant niches during periods of relatively high stress and disturbance but low competition. Further work is needed, however, to mechanistically link phenology to plant invasions and to build towards more accurate and specific predictions of how climate change may promote invaders in the future. Below we outline what we consider the major questions impeding robust predictions in this area.

### How do longer-term properties of a system's climate affect phenological invasions?

Climate change represents a long-term climate trend overlaid onto already complex climates of most ecosystems, including a climate's mean, cyclical variation (e.g. seasons and multi-year cycles often driven by large-scale oscillations) and extremes. Thus, a coherent understanding of how species and communities will shift with climate change requires consideration of more than shifts in the timing and magnitude of temperature and precipitation.

Furthermore, such a coherent picture of how species respond to shifts in temperatures and precipitation will include a focus not just on mean or aggregated climate metrics, but also on extreme events. An example of the importance of this comes from attempts to understand the correlation between phenology and performance with climate change. Several studies show that species that tend to advance with warming also tend to increase in abundance or performance (Cleland *et al.* 2012), including invasive species (Willis *et al.* 2010; Chuine *et al.* 2012); yet other work shows that the early-season (native) species most sensitive to climate are those that suffer the greatest performance losses with climate change (Inouye 2008). Such conflicting results can be better understood when considering the role of extreme events. In the latter study, a shift in earlier springs that did not coincide with a shift in frost dates produced the performance declines (Inouye 2008). Accurate predictions of which systems may have viable early-season open niches for invasions will require examining how much temperatures have shifted on average while also considering important spring climate events related to plant stress and disturbance, such as frost. To date, increases in frost risk with spring warming have been documented in parts of North America (Gu *et al.* 2008; Inouye 2008; Augspurger 2012), but not much of North America (Easterling *et al.* 2000), nor in Europe (Menzel *et al.* 2003; Scheifinger *et al.* 2003) or China (Dai *et al.* 2013), where shifts in freeze and frost days have occurred in step with the climate shifts driving earlier spring onset (Dai *et al.* 2013). Currently, there is little known about how early-season non-native versus native species cope with frost and frost risks. Additionally, most work on phenology has focused on temperature and related events such as frost, with little work on precipitation events.

Studies of precipitation-controlled systems must also deal with large-scale, longer-term cycles in precipitation that often dominate in such systems (e.g. El Niño in many semi-arid systems in western North America). Such longer-term cycles may be a critical consideration because native species may be adapted to these cycles. Relevant studies of population dynamics will, therefore, need to work across the relevant climate oscillation timescales and predictions will need to consider whether the oscillation may shift with climate change, as projected (IPCC 2007). Such oscillations may also be directly important to non-native species as they may dictate invasion lags and jumps (Salo 2005).

### Do invaders share the same strategies and trade-offs with phenology as native species?

Our predictions here are based on the plant traits literature that suggests several major options for species' phenological strategies, and thus how phenological traits may trade off with other traits. Thus, a critical assumption of our hypotheses is that these strategies are consistent across non-native and native species. It is possible, however, that non-native species may exhibit novel strategies, similar strategies that involve additional traits, or that they may exhibit trade-offs of different magnitudes.

Understanding these trade-offs more fully has additional predictive benefits. In particular, it would advance efforts to integrate phenology within a more holistic trait framework and enable scaling to ecosystem predictions more easily. If certain phenological strategies are more common in invaders, it would suggest a suite of plant traits that would change in concert with plant invasions and climate change. Recent work shows that temperate non-native species are more phenologically responsive to temperature than native species at some sites (Wolkovich *et al.* 2013) and that species that advance with warming also tend to increase in abundance and performance (Cleland *et al.* 2012). Together these findings suggest that future temperate ecosystems may be dominated by more phenologically plastic species. If such flexibility correlates with other traits (e.g. lower leaf lifespan or lower nutrient content in leaves), we would then predict cascading shifts in ecosystem properties such as decomposition and nutrient cycling.

### When and how does high phenological flexibility yield a competitive advantage?

One commonality across climatically diverse systems is evidence linking non-native species with high phenological flexibility. Across systems where the start of the growing season is determined by temperature (Wolkovich *et al.* 2013) or by precipitation (Wainwright *et al.* 2012), species that track the start of the season most closely are highly successful invaders. Understanding the benefits and trade-offs of high phenological tracking of environmental variables would address a fundamental question in invasion biology: if high plasticity yields a competitive advantage, why do species differ in their plasticity? One hypothesis is that species that track climate change well over the timescales for which we have data (or focus our analyses) may suffer major population losses during years of extreme climate. Alternatively, the current non-stationarity of climate may have shifted the playing field; this may mean that species that evolved in more variable environments are now often successful invaders. These hypotheses have not been tested to our knowledge. However, adaptations of bet-hedging models (e.g. Donaldson-Matasci *et al.* 2012), combined with currently available climate data, should allow basic vetting.

Studies examining the plasticity of phenology in non-native species may also want to consider how much evolutionary change following introduction has contributed to this plasticity (e.g. Sultan *et al.* 2013), and how quickly species can genotypically adjust to more static phenological cues post-invasion. Many of the studies mentioned here examine phenological shifts that are attributable to phenotypic plasticity (e.g. they are of marked perennial individuals or come from woody species known to shift leafing and flowering plastically between years with different climates), but recent studies have documented rapid evolutionary shifts in invaders, especially in phenology (e.g. Colautti and Barrett 2011; Konarzewski *et al.* 2012; Novy *et al.* 2013). Understanding how much phenological flexibility is driven by underlying plastic versus genetic shifts is important to projections of which species may become invaders—if much change is genotypic, it suggests then that predictions may be more difficult and will require knowing *a priori* how quickly phenology can evolve under new selection regimes. More studies examining invaders in their native and introduced ranges (e.g. Godoy *et al.* 2009; Matesanz *et al.* 2012) would begin to build data on how often phenologies shift with invasions and how similar or distinct invader phenologies are in their native versus introduced ranges (e.g. Wolkovich *et al.* 2013).

### How does evolutionary history influence phenological invasions?

Research from molecular ecology has consistently shown a strong genetic basis for leafing and flowering times within species (Howe *et al.* 2003; Fournier-Level *et al.* 2011); thus it may be expected that related species would share similarities in their phenologies, and possibly their phenological responses to climate. Indeed, a growing number of studies have documented significant evolutionary structure in the distribution of flowering times and sensitivities to climate change across species (Davis *et al.* 2010), including invaders (Willis *et al.* 2010; Wolkovich *et al.* 2013). A recent, more comprehensive analysis across >20 sites, from temperate to tropical Northern Hemisphere zones, shows phylogenetic structure in flowering and leafing for almost all communities studied (Davies *et al.* 2013), such that more closely related species also tend to have more similar phenologies. This structure means studies of phenology including multiple species may want to consider how much variation in phenology and related phenological traits is explained by the evolutionary distances separating species, versus other factors.

Considering phylogenetic structure is especially important in studies attempting to link phenology to invasion success and any studies looking for correlations between phenology and other traits, because species cannot be treated as statistically independent. For example, studies finding multiple non-native species with distinct phenologies compared with the native community will need to test how much this finding is driven by the phylogenetic affinity of non-native species compared with species in the native community. If non-native species are only distantly related to the native species pool, we might expect them to differ in their phenologies, irrespective of the actual traits explaining their invasion success. In addition, when looking at correlations between phenological traits and invasion, apparent trade-offs might simply reflect phylogenetic affinities if non-native species are evolutionarily distant for the native species pool. Our mechanistic explanation for invader success might, thus, be quite different depending on whether invaders are filtered on phylogenetically conserved traits versus a scenario in which they diverge from the native community following introduction. Phylogenetic methods aid in distinguishing between these two scenarios.

## Conclusions

With future climate change, invasive species are predicted to increase both in abundance and in spatial distribution (IPCC Core Writing Team *et al.* 2007; Bradley *et al.* 2010). We have outlined here a more focused framework for examining how phenology may affect plant invasions. This framework considers phenology as one factor by which plants attempt to optimally balance acquisition, allocation and loss in an environment where most systems' climates are now highly non-stationary. As increasing research builds to test and advance this framework, resource managers will in turn need to evaluate how they may use phenology in their decision making. If many species appear to gain a foothold or spread in introduced communities via phenological differences compared with the native community, it may suggest novel management practices. These could include optimally timing treatments for when only non-native species are active, or using phenological differences to identify species that may have a high potential to be invaders with climate change. Such applications will, of course, be bolstered by additional studies of phenology. In particular, further work is needed to understand how phenology correlates with and is constrained by other traits, whether this varies between different climate regimes, functional groups and clades, and whether non-native species appear to face the same constraints to their phenologies as native species.

## Sources of Funding

E.M.W. was supported by the Natural Sciences and Engineering Research Council of Canada's Collaborative Research and Training Experience Program (NSERC CREATE) in biodiversity research through the Biodiversity Research Centre at the University of British Columbia (Vancouver, BC, Canada).

## Contribution by the Authors

E.M.W. and E.E.C. contributed ideas and concepts and edited the manuscript. In addition E.M.W. conceived of and wrote the manuscript and designed and created all figures.

## Conflicts of Interest Statement

None declared.
